# Systematic review of lower urinary tract symptoms occurring with pelvic organ prolapse

**DOI:** 10.1080/2090598X.2019.1589929

**Published:** 2019-04-03

**Authors:** Anne P. Cameron

**Affiliations:** Department of Urology, University of Michigan, Ann Arbor, MI, USA

**Keywords:** Overactive urinary bladder, pelvic organ prolapse, lower urinary tract symptoms

## Abstract

**Objective**: To review lower urinary tract symptoms (LUTS), which include a large variety of bladder complaints, in women with simultaneous pelvic organ prolapse (POP).

**Methods**: This article is a systematic review of the current literature on LUTS occurring simultaneously with POP following the Preferred Reporting Items for Systematic Reviews and Meta-Analyses (PRISMA) methodology.

**Results**: The prevalence of both conditions is high, but they occur more frequently together than can be explained by chance. It appears that POP is in some women causative of overactive bladder (OAB) symptoms, as in many women correction of the POP resolves the bladder symptoms and small studies of women with detrusor underactivity also demonstrate resolution of symptoms. The most plausible explanation for the relationship is that POP causes bladder outlet obstruction, which results in excess bladder irritability or poor contractility. However, not all women have resolution of their OAB symptoms and some women develop them *de novo* after POP repair, so this explanation requires more in depth study.

**Conclusions**: Women with both LUTS and symptomatic POP should probably have their POP targeted, as its reduction either via surgery or pessary can correct the LUTS. However, no studies have addressed asymptomatic POP, so it is unclear if treating POP in these instances is of benefit.

**Abbreviations:** BOO: bladder outlet obstruction; DO: detrusor overactivity; DU: detrusor underactivity; OAB: overactive bladder; P_det_Q_max_: detrusor pressure at maximum urinary flow; POP: pelvic organ prolapse; PVR: post-void residual urine volume; RR: relative risk; SUFU: Society of Urodynamics, Female Pelvic Medicine and Urogenital Reconstruction; UDS: urodynamic studies; (S)(U)UI: (stress) (urgency) urinary incontinence

## Introduction

LUTS include a large variety of bladder complaints. These can be grouped into storage symptoms of urgency with or without urgency urinary incontinence (UUI), frequency and nocturia, which is commonly referred to as overactive bladder (OAB) [1]. These symptoms are very common in women, occurring in 15% of women aged 20–29 years and these become more prevalent with age, with 21% of women aged >70 years having OAB [2]. Other LUTS include emptying problems such as straining to void, hesitancy, sense of incomplete emptying and slow stream, but these are more common in men as prostate growth results in relative urethral obstruction, but are still present in a significant number of women.

Common varieties of UI are UUI, stress UI (SUI) and mixed UI. Mixed UI is by far the most prevalent, with 57% of incontinent women having this type of UI [3], which is both UUI and SUI occurring together [3]. Unfortunately, this variety of UI also is reported by patients to be more bothersome that pure SUI or UUI [3].

Pelvic organ prolapse (POP) includes cystocoele, which is an anterior vaginal wall prolapse; rectocoele a posterior vaginal prolapse; apical prolapse of the vaginal cuff after hysterectomy or uterine prolapse; enterocoele and perineal descent [4]. It is an extremely prevalent problem, which has been reported to affect 50% of parous women [5]. However, a lower grade of POP, where the prolapse is above the hymen is typically asymptomatic. Highlighting the impact that this problem presents to women and society as a whole, 11% of women will have surgery for POP or UI by the age of 80 years [6].

For example, in the Boston Area Community Health (BACH) study, one in 10 adults developed LUTS at the 5-year follow-up, and symptoms were significantly more prevalent in women and non-White minorities [7], with a sharp increase with age. With an ageing USA population, the high prevalence of LUTS also lends an enormous economic burden to the healthcare system. Ganz et al. [8] estimated that, in 2020, national costs of OAB symptoms alone will exceed $82 billion (American dollars). Importantly, LUTS also adversely affect mental and physical quality of life [9–11].

## Methods

**Figure 1.**
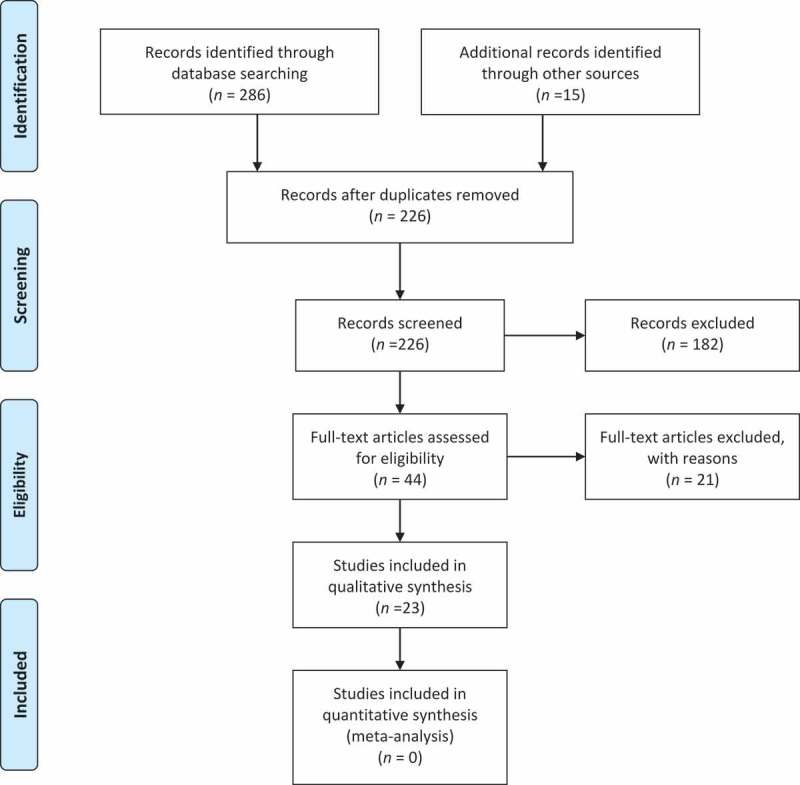
PRISMA flow chart of studies selection.

The MEDLINE and Cochrane databases were searched for original articles and systematic reviews including only adult human female subjects with the search terms ‘pelvic organ prolapse’ and ‘urgency incontinence’ or ‘detrusor overactivity’ or ‘detrusor underactivity’, from 2003 to 2018. Extra articles were included that were referenced in these original articles. Articles focusing on the co-existence of LUTS and POP were excluded if they did not have a reference group without POP. Articles focusing on the correction of POP and the subsequent resolution of LUTS were only included if they did not have a concomitant SUI surgery. Articles whose primary lower urinary tract endpoint was SUI were excluded. Case reports, non-systematic reviews and non-human studies were excluded. The literature review and article selection was conducted in accordance with Preferred Reporting Items for Systematic Reviews and Meta-Analyses (PRISMA) methodology [12] (Figure 1).

## Results

In all, 286 articles were identified in the original search with 15 additional articles of interest found via references in the articles that were read. Overall, 75 articles were duplicates and 125 articles were excluded based on screening the title for a total of 101 abstracts. Of the 101 abstracts, 25 were duplicate studies with more than one publication, 32 on review did not meet the inclusion criteria, and 44 full-text articles were assessed. Of the 44 articles, 21 were excluded for lack of a reference group or simultaneous SUI surgery for a total of 23 articles included. There was not sufficient data for a new meta-analysis beyond what has already been reported [13].

### How often do they occur together?

Given that both of these conditions are very prevalent amongst women it would be clear that they would occur together frequently purely by chance, but if one is potentially causative of the other one would expect them to occur together more often than by chance.

In a meta-analysis by De Boer et al. [13], they reviewed the prevalence of OAB in relation to POP in community-based studies. There were four studies that included a large population (12 514 women of which 1071 women had a POP) and assessed the symptoms of UUI or general OAB symptoms via questionnaire. The relative risk (RR) was calculated by dividing the frequency of OAB with POP by the frequency without POP. All four studies had very consistent results and showed a higher prevalence of OAB symptoms with POP than without POP. This review also assessed hospital-based studies and there were five studies that allowed comparison. Again the prevalence of OAB symptoms was greater in the patients with POP than in the patients without POP with a RR varying from 1.2 to 3.4.

Looking at all of these studies together the prevalence of UUI varied from 22% to 88% amongst women with POP compared to women without POP, where only 3.9% to 64% had UUI. All but one of the reviewed studies reported a significant difference in UUI between POP and non-POP, and the RR of OAB amongst patients with POP being as high as 5.8 in one large study with >5000 women, with the range of RRs of OAB with POP of 1.2–5.8 [14].

### Theories as to why they would co-occur

It is unknown exactly why LUTS would occur simultaneously with POP in such a large percentage of women, but based on pathophysiology and anatomical knowledge there exists no plausible explanation for why LUTS would cause POP. Also, none of the treatments for LUTS including anticholinergics, β_3_-adrenoceptor agonist, botulinum toxin, percutaneous tibial nerve stimulation or sacral neuromodulation, has ever resolved POP. However, there exist several plausible explanations for why POP could cause LUTS. This knowledge at least answers the ‘chicken or the egg first’ question, and POP came first.

POP, especially at higher grades, can cause bladder outlet obstruction (BOO). There exists an overall agreement that POP-induced BOO may trigger bladder changes resulting in OAB symptoms [13]. This is corroborated by the information that correction of POP can unmask SUI [15] by relieving BOO or how women notice a decrease in UI symptoms as their POP progresses likely a result of increasing BOO. BOO can cause bladder irritation and re-modelling of the detrusor in a similar fashion that BPH-related BOO affects men causing LUTS.

There are three postulated theories as to why POP could lead to OAB symptoms that were described by De Boer et al. [13] in a very comprehensive review article: (i) denervation of the autonomic nerve supply to an obstructed bladder; (ii) the progressive changes due to BOO in the detrusor muscle that cause it to be more irritable and with greater instability; and (iii) changes in the spinal micturition reflex of the obstructed bladder contributing to increased sensitivity or detrusor overactivity (DO).

Other possibilities are that the distended anterior vaginal wall stretches the bladder causing misfiring of stretch receptors in the urothelium releasing neurotransmitters such as acetylcholine and ATP [16,17]. These are sensed by neurones in the urothelium triggering bladder contractility.

However, these conditions may be independent and also be the result of a common aetiology such as pelvic floor dysfunction or trauma from childbirth or simply ageing. This may be the case in some women, especially those who do not get OAB relief with correction of their POP, but for those women who do get resolution of their OAB with a POP correction either surgically or with a pessary, the POP was likely the cause. However, it is very difficult to ascertain who these women are.

Why women would have detrusor underactivity (DU) occurring simultaneously with POP has been less well studied. Clearly if BOO is the culprit then its resolution will improve voiding symptoms but why detrusor contractility would improve is unclear [18].

### Does worse POP lead to worse OAB?

The relationship between the stage of POP and OAB is not clear and not well studied. In contrast to what might be expected, Burrows et al. [19] found that urgency and UUI occurred more often in women with a less advanced POP overall. This is not an isolated finding, with another study concluding the same utilising ultrasonography in which women with a lower grade of prolapse bladder descent were more likely to have UUI [20].

In contrast, Miranne et al. [21] found that more women with stage 3–4 POP (35%) had urodynamic DO than women with lower grade 1 or 2 POP (17%). Regardless of the stage of POP after repair, OAB symptoms of urgency and frequency improve similarly in women with stage 1 or 2 POP compared to women with stage 3 or 4 [22].

### Resolution of LUTS after POP repair

The most compelling evidence for the causal relationship between POP and OAB is that correction of POP cures the patient’s LUTS.

Surgical correction of the POP is not necessary to improve symptoms simply reduction of the POP, as shown by ring pessary fittings in women with POP. Women fitted with a pessary had a 38% improvement in urgency and 26% improvement in UUI at 4 months [23]. In another study of women with UUI and POP, there was a 46% improvement in symptoms after successful pessary fitting [24].

In one study with 109 women, where 73% had a successful fitting at 3 months, 97% of women with obstructive symptoms reported improvement and UUI improved in 77%. Urinary flow rate and residual urine improved but vaginal discharge was reported by 44% of women at 3 months and 10% developed vaginal ulcers [25]. Pessaries can also be used as an excellent diagnostic tool to see how a patient’s symptoms will resolve after POP repair and can be considered a useful tool in decision-making before embarking on a more invasive surgical option.

### Results of POP surgery

De Boer et al. [13] compiled 12 studies that assessed OAB symptoms before and after POP repair, and only included those cases without concomitant SUI surgery, which allows assessment of the effectiveness of POP alone on LUTS. Follow-up varied between 2.5 and 60 months, and most studies reported a ≥ 90% resolution. The RR of resolution was calculated as the frequency of preoperative symptoms divided by the postoperative symptoms. All but one study reported a RR of improvement >1.0 with results ranging between 1.1 and 10.3.

OAB symptoms improve regardless of surgical approach. In a group of elderly women with stage 3 or 4 POP, who underwent colpocleisis, there was a significant reduction in urgency and frequency at 1 year after surgery, with no difference compared with women who underwent reconstructive type vaginal suspensions [26].

### Risk factors for persistence of OAB after surgical repair

Several articles have attempted to determine preoperative risk factors for persistence of OAB symptoms after POP repair. The most recent Cochrane review on POP surgical management reports new or *de novo* OAB symptoms in 12% of women in nine trials [27].

Fletcher et al. [28] reported a reduction of UUI of 49% and a 74% reduction in difficulty in voiding in a group of women undergoing anterior repair. They found that persistent UUI was significantly related to higher preoperative detrusor pressure at maximum urinary flow (P_det_Q_max_). They did not find any relationship with symptom improvement or resolution with pre-surgical POP severity or the presence of DO. The higher P_det_Q_max_ further supports the theory that POP causes BOO resulting in greater DO leading to OAB symptoms, and that resolution of the BOO can improve the symptoms but may also lead to irreversible changes in the detrusor that lead to persistent bladder overactivity or symptoms.

In a more recent study, amongst 174 women who underwent transvaginal mesh repair of their POP, 49 had preoperative UUI and after the repair 10 (20.4%) had persistent UUI and 19 (38.8%) developed *de novo* SUI. Patient factors that predicted persistent UUI included preoperative urodynamic studies (UDS) findings of BOO, maximum cystometric capacity of <300 mL, bladder trabeculation, and duration of symptoms of >5 years [29], which is not surprising as it is hypothesised that prolonged BOO can lead to irreversible detrusor changes [30].

DO in 63 women undergoing transvaginal mesh repair persisted in 19 of these women, but symptomatic urgency and UUI was only present in less than half of these women before surgery (42.9% and 41.3%, respectively). Predictors of DO persistence included: preoperative UDS findings of BOO, elevated post-void residual urine volume (PVR), and concomitant sacrospinous ligament fixation [31]. In another study of 53 women undergoing transvaginal mesh repair of their POP, where the rate of OAB improvement was 66%, pre-procedure resolution of DO on UDS after POP reduction had the greatest disappearance or improvement in DO after surgery [26].

In another similar study, where 245 women had POP stage 3 or 4 and UDS finding of DO, 24.5% had persistent DO after POP repair (most were transvaginal mesh). Of the 1202 women without preoperative DO, 3.5% developed *de novo* DO. Preoperative independent predictors of persistent or *de novo* DO included: age >65 years, neurological disease such as Parkinson’s, BOO or elevated PVR >200 mL [32].

However, one must remember that the relationship between DO and OAB is not straightforward, and the UDS finding of DO is only present in 54% of women with OAB symptoms and in the reverse situation only 28% of women with DO have OAB symptoms [33].

### Medical therapy for LUTS

As stated above medical therapy for LUTS does not correct the POP, but antimuscarinics and β_3_-adrenoceptor agonists are considered second-line therapies for OAB according to the AUA/Society of Urodynamics, Female Pelvic Medicine and Urogenital Reconstruction (SUFU) OAB guidelines [34].

However, in one study, women with POP and OAB treated with tolterodine had reduced effectiveness compared to those without POP [35]. As there is compelling evidence that POP repair resolves OAB in a large percentage of women, probably via correction of BOO and that medical therapy for OAB has limited effectiveness with known side-effects and poor long-term adherence, and that delay in treatment is a risk factor for persistent OAB, it would seem reasonable to recommend medical therapy only as a temporary management plan whilst awaiting definitive therapy or as a treatment for persistent or *de novo* OAB after POP repair.

### Who should get UDS before POP repair?

Preoperative DO is not necessarily predictive of postoperative UUI or urgency, which is not surprising as DO correlates only weakly with UUI or urgency. As this finding does help prognosticate postoperative outcomes there is very little value in routine preoperative pressure–flow UDS in women undergoing POP repair. However, there is value in patients with recurrent UTIs, hydronephrosis or concomitant neurological disease, and these need to be considered on a case-by-case basis for risk stratification, especially if an anti-incontinence procedure is planned [36]. Clearly patients should be screened for SUI or occult SUI with a simple cystometric examination with their POP reduced [37]. If UDS are done according to AUA/SUFU guidelines [38] they should be performed with the POP reduced to assess for detrusor dysfunction and this manoeuvre can distinguish between BOO and DU.

### Bladder trabeculation

Amongst male patients trabeculation and hypertrophy of bladder muscles can develop due to BOO particularly caused by benign prostatic enlargement [39]. Over aggressive anti-UI procedures and severe POP are some of the most common causes of BOO in women [40], but the relationship between BOO and trabeculation is less clear. In one study [41], trabeculation was assessed via cystoscopy and graded on the modified grading systems proposed by El Din et al. [39] from 0 (none) to 4 (severe with diverticula) in 308 women with stage 3 or 4 POP. Over half of the women had trabeculation, which correlated with more LUTS, DO, and urinary retention. Also, the women with trabeculation had a higher prevalence of advanced anterior POP. From these same authors severe trabeculation was a preoperative risk factor for persistent UUI after POP repair [29].

### DU

In 518 women with POP, 41% had a UDS finding of DU, defined as a bladder contractility index of <100. These women with DU, not surprisingly, had higher PVRs and voiding symptoms, but lower rates of UUI and DO, and greater anterior compartment descent. After POP repair, women both with DU and without had similarly improved voiding symptoms, suggesting that the women with DU actually had BOO relief as the mechanism for their improvement [42].

In 49 women with stage 3 or 4 POP with DU (defined as a P_det_Q_max_ of <10 cmH_2_O and a Q_max_ of <12 mL/s), following pelvic reconstructive surgery the subjective cure rate of DU was 76% with a negative response to the question ‘Do you experience difficulty emptying your bladder?’, and 47% had resolution of the DU on UDS, with 57% recovering bladder contractions that were absent before surgery [18].

## Conclusions

OAB and POP occur commonly together in women and the connection between these two conditions is not perfectly clear. BOO due to the POP accompanied by the resulting bladder muscle changes seem to be the most plausible explanation of how POP could cause OAB, and explains why correction of POP resolves the OAB symptoms in a large percentage of patients. This explanation does not align with the notion that more severe POP (which causes more BOO) is not necessarily associated with worse OAB. Also, a substantial percentage of women do not have resolution of their OAB after POP repair, possibly because of irreversible bladder changes due to long-term BOO or perhaps these are independent conditions in some women, as OAB does occur in the absence of POP or BOO in a large number of women. DU is also often resolved with POP repair and given that there are few options for this condition POP reduction should also be there first-line therapy for this condition in women with co-existing POP.

**Figure 2.**
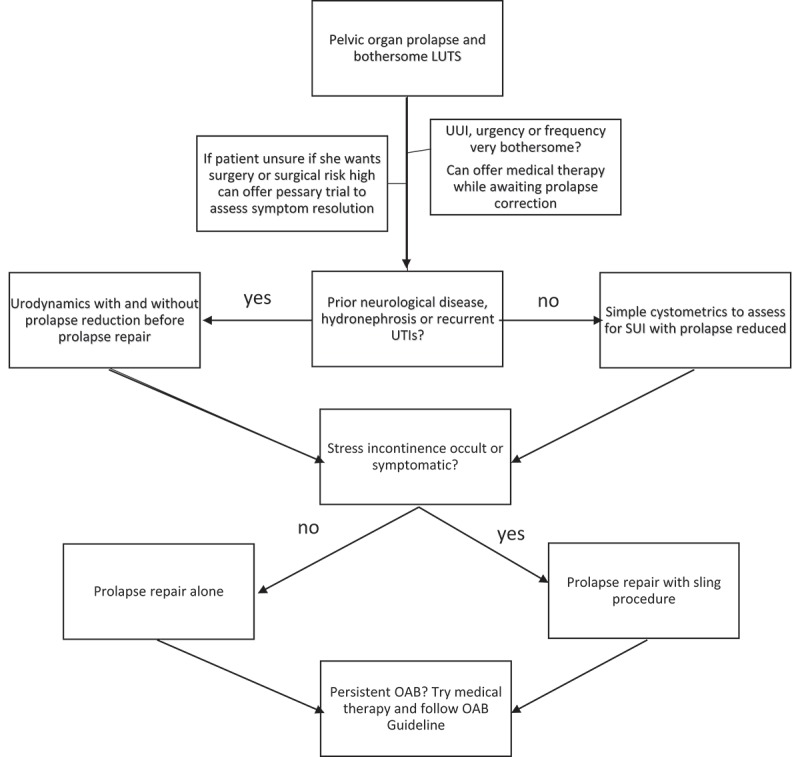
Flow chart of treatment options for women with POP and bothersome LUTS.

It would seem prudent to offer POP reduction either surgically or with a pessary to any woman with OAB and POP as a first option in their treatment, as this may be a unifying diagnosis and a single procedure could correct both (Figure 2). UDS are not absolutely necessary before surgery except in the case of pre-existing neurological disease, hydronephrosis or recurrent UTIs, as the presence of DO, DU or BOO does not change surgical planning. However, simple cystometrics with POP reduction are needed before surgery to assess for occult SUI and to counsel patients on their risk of postoperative SUI to help them decide on a concomitant sling procedure.

Women with bladder trabeculation, a longer duration of OAB symptoms, age >65 years, neurological disease, elevated PVR of >200 mL, and greater degrees of BOO have a lower chance of OAB resolution after POP repair. However, none of these risk factors substantially lowered the chance of resolution, so are not contraindications to offering POP repair.
